# Characterization of Thermostable Cellulase from *Bacillus licheniformis* PANG L Isolated from the Himalayan Soil

**DOI:** 10.1155/2023/3615757

**Published:** 2023-08-31

**Authors:** Manita Shyaula, Sunil Regmi, Deegendra Khadka, Ram Chandra Poudel, Agni Dhakal, Devesh Koirala, Jaishree Sijapati, Anjana Singh, Jyoti Maharjan

**Affiliations:** ^1^Central Department of Microbiology, Tribhuvan University, Kirtipur, Kathmandu, Nepal; ^2^Nepal Academy of Science and Technology, Khumaltar, Lalitpur, Nepal

## Abstract

This study aimed to isolate, purify, and characterize a potential thermophilic cellulase-producing bacterium from the Himalayan soil. Eleven thermophilic bacteria were isolated, and the strain PANG L was found to be the most potent cellulolytic producer. Morphological, physiological, biochemical, and molecular characterization identified PANG L as *Bacillus licheniformis*. This is the first study on the isolation of thermostable cellulase-producing *Bacillus licheniformis* from the Himalayan soil. This bacterium was processed for the production of cellulase enzyme. The optimum conditions for cellulase production were achieved at 45°C after 48 h of incubation at pH 6.5 in media-containing carboxymethyl cellulose (CMC) and yeast extract as carbon and nitrogen sources, respectively, in a thermo-shaker at 100 rpm. The enzyme was partially purified by 80% ammonium sulphate precipitation followed by dialysis, resulting in a 1.52-fold purification. The optimal activity of partially purified cellulase was observed at a temperature of 60°C and pH 5. The cellulase enzyme was stable within the pH ranges of 3–5 and retained 67% of activity even at 55°C. Cellulase activity was found to be enhanced in the presence of metal ions such as Cd^2+^, Pb^2+^, and Ba^2+^. The enzyme showed the highest activity when CMC was used as a substrate, followed by cellobiose. The *K*_*m*_ and *V*_max_ values of the enzyme were 1.8 mg/ml and 10.92 *μ*g/ml/min, respectively. The cellulase enzyme obtained from *Bacillus licheniformis* PANG L had suitable catalytic properties for use in industrial applications.

## 1. Introduction

Cellulose is a fibrous, tough, crystalline, and linear polymer of D-glucose units linked by *β*-1, 4-glycosidic bonds [1, 2]. It is a major component of plant material and the most abundant renewable source of energy [3, 4]. This cellulose is of major economic value in developing methods for successfully treating and using cellulosic wastes as cheap carbon sources [5]. Cellulases, a group of glycosyl hydrolases, can efficiently hydrolyze cellulose into fermentable sugar through the synergistic action of endoglucanase, exoglucanase, and *β-*glucosidase [6, 7].

Cellulases are used in a variety of industries, including food, brewing, pharmaceuticals, pulp and paper, detergents, textiles, leather, waste treatment, feed, and agriculture [3, 8–10]. They are commonly produced by bacteria, archaea, prokaryotes, plants, animals, and fungi [11, 12]. Comparatively, bacterial species have higher growth rates, high enzyme thermostability, better expression systems, and resistance to extreme environments [13, 14].

Recently, thermophilic bacteria have attracted a lot of attention as a source of cellulolytic enzymes. The hydrolysis of cellulose by thermophiles has various advantages such as greater stability, increased specific activity, inhibition of microbial growth, and easier mass transfer [15–17]. Thermophilic cellulose-degrading bacteria have been isolated from diverse sources such as soil [18], compost systems [19], goldmines [20], wastewaters [21], and extreme environments such as hot springs [22, 23]. Several thermophilic *Bacillus* strains, including *B. vallismortis* RG-07 [24], *Bacillus* sp. SMIA-2 [14], *Bacillus* and *Geobacillus* strains [16], and *B. halodurans* CAS 1 [25], have been reported for cellulolytic activities.

The Himalayas are highly diversified regions and have multiple stress conditions such as temperature fluctuations, excessive UV exposure, nutrient scarcity, and low oxygen and air pressure [26–28]. The evolution of microorganisms in response to these pressures has given rise to their unique biochemical and physiological diversity with innovation and unique traits [29]. Therefore, the present study was conducted to isolate and optimize the medium for potential thermostable cellulase-producing bacteria and to purify and characterize the produced enzyme according to various parameters.

## 2. Materials and Methods

### 2.1. Isolation of Thermophilic Bacteria

The soil samples were collected from three different areas of the Solukhumbu District, Nepal, namely, Pangboche (altitude 3450 m, latitude 27°51.426 N, and longitude 86°47.640 E), Lobuche (altitude 4960 m, latitude 27°57.269 N, and longitude 86°48.89 E), and Makalu Barun National Park (altitude 3700 m 27°39.29 N and longitude 87°45.52 E). One gram of soil sample was suspended in 9 ml of sterile Milli-Q water and serially diluted under sterile conditions. The diluted cultures were evenly spread on nutrient agar (NA) plates and incubated at 55°C for 24 h. The pure cultures on the NA medium were transferred to a freshly prepared NA slant with 20% glycerol and stored at −20°C [30].

### 2.2. Assessment of Enzymatic Production

The pure isolates were streaked on CMC agar plates and incubated at 55°C for 24 h [31]. The plates were stained with 0.1% congo-red solution for 15 min and washed with 1 M NaCl for destaining [32]. The bacterial isolates were also screened for other industrially important enzymes like amylase, lipase, caseinase, pectinase, and gelatinase [33–36]. The cultures were inoculated in nutrient broth (NB) at 55°C. For enzyme production, 1 ml of the culture was inoculated in 10 ml of CMC broth and incubated overnight at 55°C under shaking conditions at 100 rpm [37].

### 2.3. Morphological, Physiological, and Biochemical Characterization of the Isolates

The selected cellulase-producing bacterial isolate was identified by performing Gram staining and several biochemical and carbohydrate utilization tests [38].

### 2.4. Amplification of the 16S rDNA Gene Using PCR

The DNA was extracted and quantified by NanoDrop. The 16S rDNA gene was amplified by PCR from the genomic DNA of the strain using universal primer pair fD1 (5′-AGAGTTTGATCCTGGCTCAG-3′) and rP2 (5′-ACGGCTACCTTGTTACGACTT-3′). Amplification of DNA was carried out under the following conditions: denaturation at 94°C for 2 min followed by 35 cycles of 94°C for 1 min, 55°C for 1 min, 72°C for 2 min, and a final extension at 72°C for 10 min. Amplified PCR products of bacterial isolates were analyzed by electrophoresis on 1% agarose gel at 80 V for 30 min, stained with ethidium bromide, and visualized in a gel documentation system [39]. The PCR products were purified using exonuclease shrimp alkaline phosphatase (Exo-SAP) kit protocol and then sequenced by using BigDye Terminator Cycle Sequencing kit (Applied Biosystems, CA). The sequencing results were compared using the basic local alignment search tool (BLAST) on NCBI and 16S rRNA gene sequence homology evaluation carried out using GenBank data. A phylogenetic tree was constructed using MEGA 6.0 [40].

### 2.5. Optimization of Culture Conditions on Cellulase Activity

The optimization of various physicochemical parameters of growth conditions was carried out for maximum cellulase production. The effect of a single parameter was determined at a time by keeping the rest of the parameters constant. The major parameters and their effects on cellulase production were determined by measuring the incubation period (24–120 h), pH (3–11), temperatures (30–70°C), and various agitation speeds (static, 50, 100, 150, and 200 rpm). Various carbon sources tested included CMC, xylose, maltose, glucose, fructose, starch, sucrose, and cellobiose. Different types of nitrogen sources such as potassium nitrate, ammonium sulphate, tryptone, ammonium nitrate, ammonium chloride, peptone, yeast extract, and beef extract were examined for their effects on growth and enzyme production. For each step, the enzyme activity was assayed to determine the optimal yield [41].

### 2.6. Extraction of Crude Enzyme

The isolated bacterial strain was cultured in CMC broth and incubated at 45°C for 48 h under shaking conditions (100 rpm). The culture was centrifuged at 10,000 rpm for 10 min at 4°C, and the supernatant was used as a crude enzyme for cellulase activity assay and partial purification [22].

#### 2.6.1. Cellulase Assay

Cellulase activity was determined by measuring the amount of reducing sugar liberated from CMC using the 3, 5-dinitrosalicylic acid (DNS) method [42]. The enzyme assay mixture was prepared by mixing 500 *μ*l of crude enzyme solution with 500 *μ*l of 1% (w/v) CMC dissolved in 0.1 M phosphate buffer at pH 7. The mixtures were incubated at 45°C for 15 min. The reactions were stopped by adding 1 ml of DNS reagent. All the mixtures were heated in boiling water at 100°C for 5 min for color development, and the optical density was measured at 540 nm. All of the cellulase assays were performed in triplicate. The enzyme activity was determined by using a calibration curve for glucose. One unit (U) of the enzyme activity is defined as the amount of enzyme that releases 1 *μ*mol of glucose per minute [43].

### 2.7. Partial Purification of Cellulase

Partial purification of the crude enzyme was carried out by fractionation using ammonium sulphate (20–80%) followed by dialysis The crude enzyme was precipitated with ammonium sulphate overnight at 4°C in a magnetic stirrer and centrifuged at 10,000 rpm for 15 min at 4°C to collect the pellets. The pellets were resuspended in a small amount of 0.1 M phosphate buffer, pH 7, and dialyzed overnight for 24 h at 4°C against the same sample buffer by using snakeskin-pleated dialysis tubing [22]. Protein concentrations in the crude sample were estimated by using the biuret method with bovine serum albumin as a standard [44].

### 2.8. Characterization of the Enzyme

#### 2.8.1. Effect of Temperature and pH on Enzyme Activity and Stability

The optimum temperature of the enzyme was determined by incubating the mixture of the enzyme and 1% CMC in 0.1 M phosphate buffer and pH 7 for 15 min at different temperatures ranging from 30°C to 90°C. The heat stability of the enzyme was determined by incubating the enzyme in 0.1 M phosphate buffer and pH 7 for 60 min at temperatures ranging from 30°C to 90°C for a period of 1 h. The residual activity of each sample for the hydrolysis of CMC was then calculated under assay conditions [22].

The optimum pH of the cellulase was determined by incubating the mixture of the enzyme and 1% CMC in the presence of buffers: 0.1 M acetate buffer (pH 3–5), 0.1 M phosphate buffer (pH 6–8), and 0.1 M glycine NaOH buffer (pH 9–11). The reaction mixtures were incubated at 60°C for 15 min. For the determination of pH stability, the enzyme was incubated in different buffers at different pH ranges (pH 3–11) for 1 h at 60°C [22].

#### 2.8.2. Effect of Incubation Time, Various Metal Ions, Different Substrates, and Time Stability on Enzyme Activity

The optimum incubation time of the enzyme was determined by incubating the mixture of the enzyme and 1% CMC in 0.1 M phosphate buffer and pH 7 at optimum temperatures (60°C) for 15, 30, 45, 60, 75, and 90 min. The enzyme activity at each incubation time was monitored using the DNS assay [22]. The effect of various metal ions on the enzyme activity was determined by the presence of Na^+^, K^+^, EDTA, Mn^2+^, Ca^2+^, Ba^2+^, Fe^2+^, Fe^3+^, Zn^2+^, Mg^2+^, Ni^2+^, Co^2+^, Pb^2+^, and Cd^2+^ metal ions. The concentration of each metal ion was 10 mM in 0.1 M acetate buffer pH 5 [22].

The specificity of the cellulase substrate was determined by testing different substrates, namely, CMC, filter paper, and cellobiose substrate [45]. The enzymes were kept at 25°C, 4°C, and −20°C for 25 days, and the residual cellulase activities were measured at intervals of five days [46].

#### 2.8.3. Enzyme Kinetics

The *K*_*m*_ and *V*_max_ values were determined by plotting velocity against substrate concentration CMC (5–30 mg/ml). The data were plotted and kinetic constants were calculated. Calculations were also performed using the Lineweaver–Burk plot [47].

### 2.9. Data Analysis

All the measurements were conducted in triplicate, and the values were reported as mean ± S.D. GraphPad Prism 8.4.3 and MS-Excel 2013 were used for data analysis and graphical illustrations.

## 3. Results

### 3.1. Isolation and Screening for Cellulase Production

Eleven thermophilic bacterial strains were isolated from the Himalayan soil. All the isolates produced cellulase, amylase, gelatinase, and lipase enzymes. Nine isolates produced caseinase, while pectinase was not reported in any of the isolates. The isolate coded PANG L showed maximum cellulase activities (0.044 ± 0.004 U/ml) and was processed further (Table 1).

### 3.2. Characterization and Identification of PANG L Bacterial Isolate

**Figure 1:**
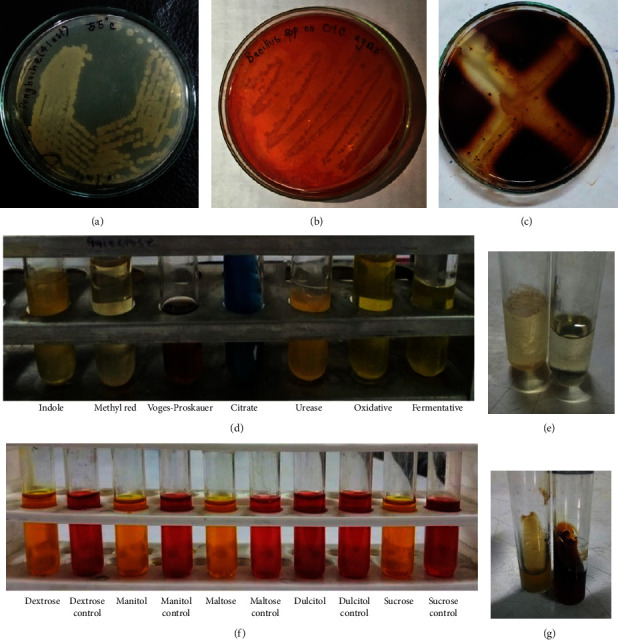
*B. licheniformis* PANG L and its characteristics: (a) colonies of *B. licheniformis* PANG L on NA, (b) cellulase activity of *B. licheniformis* PANG L on CMC agar, (c) amylase activity, (d) biochemical test, (e) growth on 10% NaCl, (f) substrate fermentation test, and (g) esculin test.

**Figure 2:**
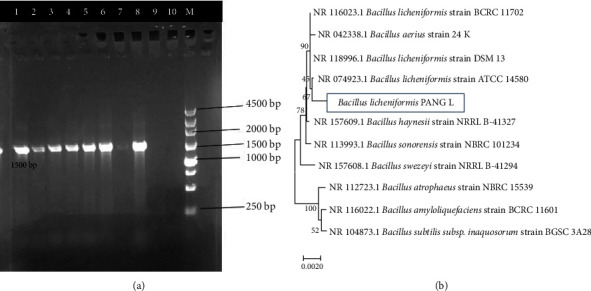
Characterization of the *B. licheniformis* PANG L isolate: (a) visualization of PCR-amplified *B. licheniformis* PANG L 16S rDNA bands on agarose gel, lanes 1, 2, 3, 4, 5, 6, 7, and 8: sample, lane 9: negative control and lane M: DNA ladder (Takara Dye Plus of 250 bp) and (b) phylogenetic tree for *B. licheniformis* PANG L and other related strains. The tree was obtained using the 16S rDNA sequence retrieved from the database by using the neighbor-joining method. The bootstrap values were generated from 1000 replicates.

The morphological, physiological, and biochemical test results of PANG L are shown in Tables 2–4 as well as in Figure 1. The concentration of DNA for the PANG L was 269 *μ*g/ml with purity (*A*_260_/*A*_280_) value of 1.79. The amplified PCR product was l.5 Kb which was further purified and sequenced (Figure 2(a)). The 16S rDNA gene sequence of PANG L exhibited maximum homology (99%) with strain *B. licheniformis*. The 16S rDNA sequence was submitted to Gene bank with the accession number OQ455938. According to the phylogenetic tree, the isolate PANG L was closely related to the *B. licheniformis* strain ATCC 14580 (Figure 2(b)). Based on these results, the isolate was designated as *B. licheniformis* strain PANG L.

### 3.3. Optimization of *B. licheniformis* PANG L Culture Conditions and Enzyme Activity

**Figure 3:**
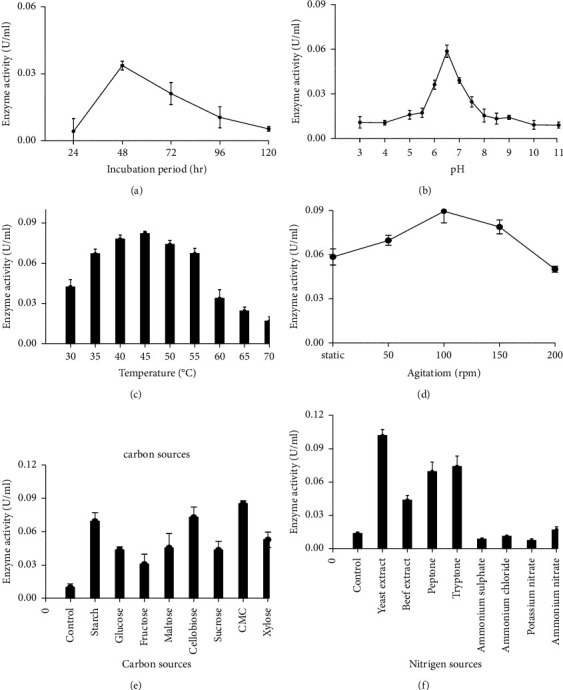
Optimization of *B. licheniformis* PANG L culture parameters and enzyme activity: effects of (a) incubation period, (b) pH, (c) temperature, (d) agitation, (e) carbon sources, and (f) nitrogen sources on cellulase production.

The optimum incubation period, pH, temperature, agitation, carbon, and nitrogen sources were determined to improve the overall growth and enzyme production (Figure 3). The enzyme-producing ability of the isolate increased with the fermentation period up to 48 h (0.034 ± 0.002 U/ml) thereafter, it declined (Figure 3(a)). The optimum enzyme production (0.058 ± 0.008 U/ml) was recorded at pH 6.5. A sharp decrease in the cellulase activity was observed below and above this pH (Figure 3(b)). The maximum cellulase activity (0.083 ± 0.001 U/ml) was found to be at 45°C (Figure 3(c)). The optimum agitation rate for higher cellulolytic enzyme production (0.089 ± 0.003 U/ml) was observed at 100 rpm while the production was the least at 200 rpm (Figure 3(d)). CMC was found to be the most suitable carbon source, which recorded a maximum enzyme activity of (0.085 ± 0.004 U/ml) followed by cellobiose (0.072 ± 0.008 U/ml) (Figure 3(e)). Among all nitrogen sources, the maximum cellulase activity (0.103 ± 0.005 U/ml) was observed when yeast extract was used as a source of nitrogen. Potassium nitrate (0.012 ± 0.002 U/ml) was found to be the least effective nitrogen source (Figure 3(f)).

### 3.4. Partial Purification of Cellulase

Ammonium sulphate precipitation of the crude enzyme was standardized, and the maximum activity was observed at 80% saturation. Therefore, 80% ammonium sulphate was used and no other concentration was applied. The crude enzyme exhibited a specific activity of 0.271 U/mg, whereas the ammonium sulphate precipitated and dialyzed enzyme showed a specific activity of 0.344 U/mg with 1.26 and 0.413 U/mg with 1.52-fold enhancement, respectively (Table 5).

### 3.5. Partial Characterization of Cellulase Enzyme

#### 3.5.1. Effect of Temperature and pH on Enzyme Activity and Stability

**Figure 4:**
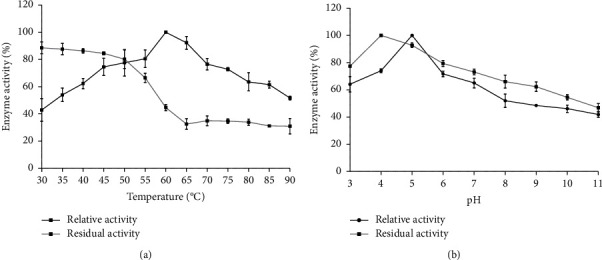
Effects of temperature and pH on cellulase activity and stability (a) temperature and (b) pH.

The optimum cellulase activity was observed at an incubation temperature of 60°C but gradually declined this temperature. Regarding the thermal stability of the cellulase enzyme, it retained 67% of its activity after preincubating at 55°C for 1 h. When the temperature was increased to 75°C, the activity reduced by 35% (Figure 4(a)). Based on the results of the pH activity and stability in Figure 4(b), it is observed that the maximum cellulase activity was maintained at pH 5 in 0.1 M sodium acetate buffer and the activity decreased as the pH increased towards alkalinity. The enzyme retained more than 70% of its activity over the pH range of 3–6. More than 50% of the activity of the cellulase was maintained at a broad pH range, ranging from pH 7 to 10 after 1 hr.

#### 3.5.2. Effect of Incubation Time, Metal Ions, Substrates, and Storage Stability on Enzyme Activity

**Figure 5:**
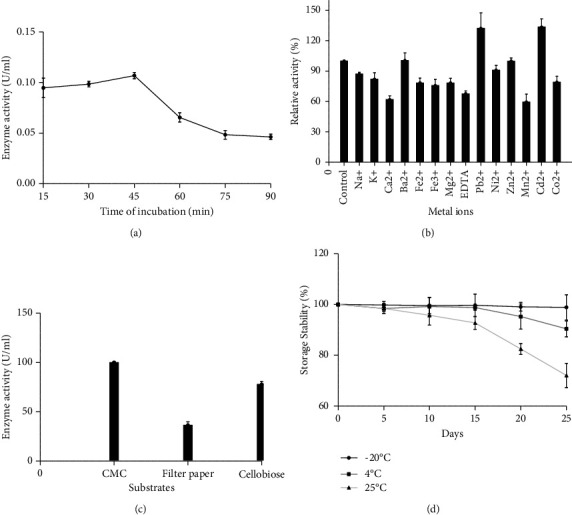
Characterization of cellulase enzyme. Effects of (a) incubation time, (b) metal ions, (c) different substrates, and (d) storage stability.

The optimum incubation time with the substrate 1% CMC in 0.1 M phosphate buffer, pH 7 was found to be 45 min. Further incubation for more than 45 min resulted in a gradual loss of the enzymatic activity (Figure 5(a)). The activity of the enzyme increased in the presence of Cd^2+^ followed by Pb^2+^and Ba^2+^, respectively, whereas the presence of manganese and calcium ions significantly decreased the activity of the enzyme (Figure 5(b)).

The enzyme showed the highest activity towards CMC (100%) and moderate activity towards cellobiose (77.48%) and the least activity towards filter paper (36.32%) (Figure 5(c)). The enzyme retained 72% of activity after storing at 25°C for 25 days, while 98.8% of the activity was recorded for the enzyme stored at −20°C. About 8% loss of activity was recorded at 4°C (Figure 5(d)).

#### 3.5.3. Enzyme Kinetics

**Figure 6:**
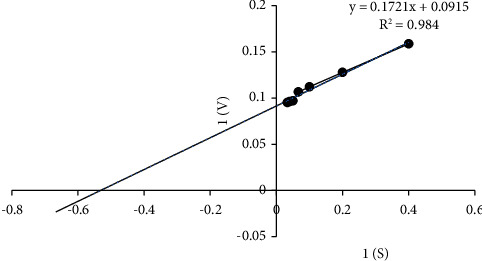
Lineweaver–Burk double reciprocal plots of cellulase from *B. licheniformis* PANG L.

Kinetic analysis with CMC revealed the *K*_*m*_ and *V*_max_ values 1.8 mg/ml and 10.92 *μ*g/ml/min, respectively, by the Lineweaver–Burk plot (Figure 6).

## 4. Discussion

The demand for microbial cellulase enzymes is continuously increasing due to their tremendous importance in the bioenergy and bioprocessing industries. The use of novel cellulase from various thermophiles could improve thermophilic cellulase production in the industrial process [48]. The present study focused on the search for high cellulase-producing thermophilic bacterial isolates from the Himalayan soil. The studies conducted by Marchant et al. [49] and Thakur et al. [50] reported thermophilic microorganisms from cold environments. Eleven isolates were able to grow at 55°C. Each thermophilic isolate produced thermostable hydrolytic enzymes such as lipase, cellulase, amylase, caseinase, and gelatinase. This showed that soil-derived bacterial isolates were the source of extracellular enzymes. Other studies have also reported hydrolytic activities in several thermophilic bacterial strains [51, 52]. Based on the quantitative cellulase assay, isolate PANG L exhibited higher cellulase activity among the isolated strains. The obtained cellulase activity was lower than that of other studies conducted by Ladeira et al. [14] and Kazeem et al. [53], indicating that the PANG L strain is a moderate cellulase producer. The morphological, physiological, biochemical, and molecular characterization confirmed PANG L as *B. licheniformis*. In previous studies, *B. licheniformis* has been reported to produce cellulase enzymes, including *B. licheniformis *2D55 [53], *B. licheniformis* AMF-07 [54], and *B. licheniformis* JK7 [55].

Optimization of fermentation conditions can improve cellulase production and play a significant role in an industrial bioprocess [56]. The fermentation process was carried out for up to 120 h. The results revealed that maximum enzyme production was obtained at 48 h. The slight reduction in the cellulolytic activity after 48 h might be due to either unavailability of nutrients or inhibition by toxic components present in the medium [57]. The study conducted by Shahid et al. [58] reported a similar trend in cellulase production by *Bacillus* sp. Temperature is a crucial factor that controls bacterial physiology and enzyme production ability [59]. The optimum cellulase production was obtained at 45°C. However, several other studies revealed that the cellulase production was achieved at 50°C by *Bacillus subtilis* K-18 [18] and at 40°C by *B. subtilis* [60]. In the case of pH, maximum cellulase production was observed at pH 6.5. A similar result was also found for *B. licheniformis* MVS1 by previous researchers [61].

Agitation is another factor that plays an important role in the transfer rate of nutrients and oxygen, the increased aerobic metabolism of microbes, and cell aggregate dispersion [62]. The current study showed that the maximum enzyme production was observed at an agitation rate of 100 rpm. At increased agitation, the enzyme may get inactivated due to the shearing of the bacterial cell [63]. The choice of the cheapest substrate is of great importance for the production of enzymes. CMC was found to be the best carbon source for the maximum production of cellulase, which is similar to other studies [64, 65]. This might be due to the activation of regulatory mechanisms responsible for higher cellulase production [48]. Organic nitrogen sources result in better cellulase production as compared to inorganic sources. This enhancement may be due to other nutrients and growth stimulators in the organic nitrogen source besides nitrogen [66]. The findings agreed with the results of Shahid et al. [58], who reported that organic nitrogen sources were more suitable for optimizing cellulase production by *B. megaterium* than inorganic sources. Through this successive selection of incubation time, temperature, pH, agitation, carbon, and nitrogen source, a 1.7-fold increase in the cellulase activity was obtained for the strain PANG L.

The effect of temperature on the cellulase activity was determined at different temperatures ranging from 30°C to 90°C. The maximum enzyme activity was observed at 60°C indicating that the enzyme is moderately thermostable. The findings were also consistent with those obtained from *Bacillus* strains M-9 [67], *Bacillus subtilis* [68], and *B. megaterium* BM05 [58] lower than that for *B. vallismortis* RG-07 65°C [24] and *B. licheniformis* JK7 70°C [55]. The concern of thermal inactivation of the enzyme is often experienced in industrial processes [46]. Hence, enzyme stability is a critical issue on an industrial scale. The thermal stability of the cellulase indicated that the enzyme retained 67% activity up to 55°C. Therefore, it was concluded that the enzyme was moderately thermostable and could have a promising industrial application. In the case of pH, the optimal enzyme activity was found at pH 5 representing the acidophilic nature of the enzyme. The same trend was also obtained from *Bacillus* strain M-9 [67]. On the contrary, Kim et al. [69] reported that *Bacillus* sp. HSH-910 was optimally active at alkaline pH. The enzyme showed good stability toward acidic pH ranges of 3–6. Bischoff et al. [70] reported that cellulase from *B. licheniformis* was more stable under acidic conditions. The optimum incubation time with the substrate was found to be 45 min and declined thereafter. The decrease in the cellulase activity beyond 45 min may be due to the thermal denaturation of the enzyme.

Metal ions can form complexes with proteins and other molecules related to enzymes and act as donors or acceptors of electron as structural regulators [71]. The cellulase activity was stimulated in the presence of metal ions such as Pb^2+^, Cd^2+^, and Ba^2+^. The partial inhibition was observed in Ni^2+^ and Zn^2+^. In accordance with the study conducted by Gaur and Tiwari [24], there is partial inhibition of cellulase observed in the presence of Ni^2+^ and Zn^2+^. However, the activity was strongly inhibited by Mn^2+^ and Ca^2+^ in the study reported by *Bacillus* strain [64]. The enzyme exhibited a high activity toward CMC. The substrate specificity of CMC indicates that the enzyme might be an endo-*β*-1, 4-glucanase [14]. Islam and Roy [41] also found the same characteristics of enzymes produced by *Paenibacillus* sp. The *K*_*m*_ and *V*_max_ values were found to be 1.8 mg/ml and 10.92 *μ*g/ml/min, indicating that the enzyme has a high affinity towards CMC due to its low *K*_*m*_ value. The results differ from some earlier studies in which the *K*_*m*_ value was higher at 7.2 mg/ml [45] and 1.923 mg/ml [24]. The important and desirable quality for industrial applications is the capacity to retain enzymes at room and/or refrigerated temperatures without significant loss of activity. From this study, it was observed that the enzyme was stable at room temperature for less than 10 days after which a slight decline was observed. The enzyme was stable at −20°C. This implies that the room temperature was less suitable for enzyme storage, resulting in a decrease in the enzyme activity. However, Islam et al. [72] reported that 68% of activities were retained at room temperature after 28 days for cellulase from a *Bacillus *sp.

## 5. Conclusions

A cellulase-producing bacterium, *B. licheniformis* strain PANG L, showed an optimum activity at a temperature of 60°C and pH 5, with good stability at pH ranges of 3–5, indicating its thermo-acidic nature. Considering its stability at elevated temperatures and acidic conditions, the cellulase from *B. licheniformis* strain PANG L could be desirable for bioconversion processes and industrial applications. Besides cellulase, *B. licheniformis* strain PANG L also produced important hydrolytic enzymes for various substrates such as lipids, proteins, and starch indicating its potential for various industrial applications.

## Figures and Tables

**Table 1:** Quantitative screening of cellulase enzyme.

Isolates' codes	Enzyme activity (U/ml) mean ± standard deviation
MBN A	0.028 ± 0.007
MBN B	0.030 ± 0.001
MBN CP	0.014 ± 0.008
MBN CW	0.013 ± 0.006
MBN DP	0.003 ± 0.007
MBN DW	0.042 ± 0.004
LOB P	0.019 ± 0.003
LOB W	0.029 ± 0.003
PANG P	0.016 ± 0.003
PANG W	0.026 ± 0.002
PANG L	0.044 ± 0.004

**Table 2:** Morphological and physiological characteristics.

Characteristics	*B. licheniformis* PANG L results
Cell morphology	
Shape	Rod

Colony	
Color	Pale
Margin	Irregular
Surface	Flat
Consistency	Rough
Light transmission	Opaque
Gram's staining	Gram-positive
Spore	Subterminal endospore
Motility	Motile
Anaerobic growth	Positive

NaCl	
7% NaCl	Positive
10% NaCl	Positive
Growth at different temperature	30 to 65°C

**Table 3:** Phenotypic characteristics.

Biochemical tests	*B. licheniformis* PANG L results
Catalase	Positive
Oxidase	Positive
Indole	Negative
Methyl red	Negative
Voges–Proskauer	Positive
Citrate utilization	Positive
Oxidative/fermentative	Positive
Urea hydrolysis	Negative
Nitrate reduction	Positive
H_2_S production	Negative

**Table 4:** Substrate fermentation test.

Carbohydrate utilization test	*B. licheniformis* PANG L results
Cellobiose	Positive
Dextrose	Positive
Dulcitol	Negative
Esculin	Positive
Fructose	Positive
Cellulose	Positive
Galactose	Negative
Glucose	Positive
Glycerol	Positive
Glycogen	Negative
Inositol	Negative
Lactose	Positive
Maltose	Positive
Mannose	Positive
Raffinose	Negative
Starch	Positive
Salicin	Positive
Sorbitol	Negative
Sucrose	Positive
Xylose	Positive

**Table 5:** Purification profile of cellulase from *B. licheniformis* PANG L.

Purification step	Cellulase activity (U/ml)	Protein (mg/ml)	Specific activity (U/mg)	Purification fold
Crude	0.106 ± 0.003	0.39 ± 0.04	0.271	1
80% ammonium sulphate pool	0.179 ± 0.002	0.52 ± 0.03	0.344	1.26
Dialysis	0.112 ± 0.002	0.271 ± 0.05	0.413	1.52

## Data Availability

Data related to the tables, graphs, and calculations are included in the article.

## Abbreviations

BLAST: Basic local alignment search tool
BSA: Bovine serum albumin
CMC: Carboxymethyl cellulose
DNS: 3, 5-dinitrosalicylic acid
EXO-SAP: Exonuclease shrimp alkaline phosphatase
MEGA: Molecular evolutionary genetics analysis
NA: Nutrient agar
NB: Nutrient broth
NCBI: National Center for Biotechnology Information.
